# Modulation of the Somatosensory Evoked Potential by Attention and Spinal Cord Stimulation

**DOI:** 10.3389/fneur.2021.694310

**Published:** 2021-08-03

**Authors:** Guiomar Niso, Marleen C. Tjepkema-Cloostermans, Mathieu W. P. M. Lenders, Cecile C. de Vos

**Affiliations:** ^1^McConnell Brain Imaging Centre, Montreal Neurological Institute, McGill University, Montreal, QC, Canada; ^2^Psychological & Brain Sciences, Indiana University, Bloomington, IN, United States; ^3^ETSI Telecomunicación, Universidad Politécnica de Madrid and Center for Biomedical Research Network CIBER-BBN, Madrid, Spain; ^4^Department of Neurology and Neurosurgery, Medisch Spectrum Twente, Enschede, Netherlands; ^5^Department of Clinical Neurophysiology, Institute for Technical Medicine, University of Twente, Enschede, Netherlands; ^6^Center for Pain Medicine, Erasmus University Medical Center, Rotterdam, Netherlands

**Keywords:** spinal cord stimulation, somatosensory evoked potential, neuromodulation, electro-encephalography, chronic pain, burst stimulation, attention

## Abstract

**Introduction:** Spinal Cord Stimulation (SCS) is a last-resort treatment for patients with intractable chronic pain in whom pharmacological and other treatments have failed. Conventional tonic SCS is accompanied by tingling sensations. More recent stimulation protocols like burst SCS are not sensed by the patient while providing similar levels of pain relief. It has been previously reported that conventional tonic SCS can attenuate sensory-discriminative processing in several brain areas, but that burst SCS might have additional effects on the medial, motivational-affective pain system. In this explorative study we assessed the influence of attention on the somatosensory evoked brain responses under conventional tonic SCS as well as burst SCS regime.

**Methods:** Twelve chronic pain patients with an implanted SCS device had 2-weeks evaluation periods with three different SCS settings (conventional tonic SCS, burst SCS, and sham SCS). At the end of each period, an electro-encephalography (EEG) measurement was done, at which patients received transcutaneous electrical pulses at the tibial nerve to induce somatosensory evoked potentials (SEP). SEP data was acquired while patients were attending the applied pulses and while they were mind wandering. The effects of attention as well as SCS regimes on the SEP were analyzed by comparing amplitudes of early and late latencies at the vertex as well as brain activity at full cortical maps.

**Results:** Pain relief obtained by the various SCS settings varied largely among patients. Early SEP responses were not significantly affected by attention nor SCS settings (i.e., burst, tonic, and sham). However, late SEP responses (P300) were reduced with tonic and burst SCS: conventional tonic SCS reduced P300 brain activity in the unattended condition, while burst SCS reduced P300 brain activity in both attended and unattended conditions.

**Conclusion:** Burst spinal cord stimulation for the treatment of chronic pain seems to reduce cortical attention that is or can be directed to somatosensory stimuli to a larger extent than conventional spinal cord stimulation treatment. This is a first step in understanding why in selected chronic pain patients burst SCS is more effective than tonic SCS and how neuroimaging could assist in personalizing SCS treatment.

## Introduction

Spinal Cord Stimulation (SCS) is a last-resort treatment for patients with intractable neuropathic pain in whom pharmacological and other treatments have failed. SCS is based on electrical stimulation of the nerve fibers in the spinal cord dorsal column (A-beta fibers) by an implanted electrode that is connected to an implanted pulse generator. Pain reduction occurs in the body area corresponding to the stimulated spinal segments. Conventional tonic SCS (i.e., single electrical pulses, given with a frequency of 30–120 Hz) is accompanied by tingling sensations (paresthesia). More recently developed stimulation protocols like burst stimulation (i.e., five pulses with intraburst frequency of 500 Hz, given with a frequency of 40 Hz) and other high-frequency stimulations (up to 10 kHz) are paresthesia-free while providing similar levels of pain relief (1–4). Although the absence of sensations is not necessarily preferred by all patients, it is an important improvement for research as it enables double-blind studies of SCS efficacy and mechanisms.

In addition to spinal action (5, 6) cerebral mechanisms are likely to contribute to the pain relieving effects of SCS (7–9), but this has not been thoroughly investigated yet. It has been suggested that tonic SCS normalizes thalamocortical dysrhythmia and overactivation (in the theta and low beta frequency range) in several pain processing cortical areas (10). In line with this hypothesis, a functional magnetic resonance imaging (fMRI) study indicated that conventional tonic SCS decreased connectivity between the thalamus and pain processing brain regions like the cingulate cortex, insula and sensorimotor cortex (11).

Burst SCS might even have effects on cortical regions outside the pain processing network: a resting state electro-encephalography (EEG) study in five patients who were trialing SCS, showed that compared with conventional tonic SCS, burst SCS led to increased synchronized alpha activity in the cingulate cortex and dorsolateral prefrontal cortex as well as behaviorally decreased attention to the pain. It was suggested that the analgesic effects of burst SCS are obtained by modulating both the lateral discriminatory and medial affective/attentional pain pathways (12). After a more thorough analysis of the data it was concluded that both tonic and burst SCS modulate the descending pain inhibitory system and the lateral pain pathway, but that burst SCS in addition modulates the activity in medial affective/attentional pain pathway (13).

One of the consequences of these findings reported by De Ridder et al. is that if reduction of cortical attention to pain is one of the working mechanisms of burst stimulation, burst might not only cause alterations in resting state activity, but also influences the capacity for attending and processing peripheral somatosensory input. In the present explorative study, we assessed the influence of attention on somatosensory evoked brain responses under conventional tonic SCS as well as burst SCS regime.

It has been previously reported that conventional tonic SCS can attenuate the somatosensory processing in SI, SII and the cingulate cortex [e.g., (8, 14–18)]. We expect that burst SCS will not only reduce the somatosensory evoked activity, but that it will also attenuate activity that is associated with attention to pain and that it will do that to a greater extent than conventional tonic SCS treatment. Insight in the various working mechanisms of action of burst SCS and other new SCS regimes will assist with better treatment selection, personalized SCS settings and optimized pain reduction for chronic pain patients.

## Materials and Methods

### Subjects

Twelve chronic pain patients (6 men, 6 women), on average 57 years old, all with Failed Back Surgery Syndrome (FBSS) and pain in their low back as well as one or two legs participated in this study. Those 12 patients also participated in the larger Burst evaluation study described in a previous publication (19). The Burst evaluation study was designed to study the effects of burst SCS on the perceived pain and quality of life in 40 patients who were already familiar with spinal cord stimulation. The EEG measurements were an optional addition to this larger study and about one third of the chronic pain patients volunteered to undergo the three additional EEG recording sessions. The study conformed to the Declaration of Helsinki and received approval from the Twente Ethics Committee. Written informed consent was obtained from 12 patients to additionally participate in the EEG measurements. The study was registered in the Netherlands clinical trial register (www.trialregister.nl, NTR 4479).

All 12 participating patients had three EEG recording sessions between August 2014 and March 2015 (Table 1). Ten patients were using analgesic medication (either co-analgesic medication like antidepressants and anti-epileptic drugs, or opioids, or a combination of those) but did not change their medication intake during the study. All received adjuvant treatment for their pain with conventional tonic SCS (Eon stimulator, St Jude Medical, Plano, TX, USA) for on average 2.7 years. Before they received their stimulator they already had pain for on average 11 years, but this varied largely over the participants, from 1 up to 35 years. In the year(s) prior to the present study, stimulation settings had been optimized for each individual patient. Perceived pain was scored by the patients on a visual analog scale (VAS) ranging from 0 (no pain at all) to 100 (worst pain imaginable). Prior to implantation of their SCS system, the patients had an average VAS score for pain of 79 (range: 70–90). The participating patients were good, moderate as well as poor responders to conventional tonic SCS, reflected in an average VAS score of 61, with a range varying from 17 to 90.

**Table 1:** Participants' demographics and their responses to the three different spinal cord stimulation (SCS) regimes: tonic SCS, sham SCS, and burst SCS.

**#**	**Sex**	**Age (y)**	**Most affected side**	**Pain prior to SCS (y)**	**Duration SCS (y)**	**Pain prior to SCS (VAS)**	**Pain tonic SCS (VAS)**	**Pain sham SCS (VAS)**	**Pain burst SCS (VAS)**	**Preference**
1	M	45	Left	3	0.5	80	90	71	65	Burst
2	M	55	Right	4	3	80	67	54	30	Burst
3	F	46	Left	17	2.5	90	67	60	40	Burst
4	F	45	Left	1	3	80	85	52	46	Burst
5	M	61	Right	4	1.5	80	55	51	49	Tonic
6	F	41	Left	18	2.5	80	67	67	79	Tonic
7	M	64	Right	3.5	2.5	80	64	54	17	Burst
8	F	66	Left	15	1	70	17	21	21	Tonic
9	M	66	Left	35	2.5	80	60	62	30	Burst
10	F	70	Left	5	6.5	80	61	50	69	Sham
11	M	65	Left	6	3	70	53	72	77	Tonic
12	F	65	Left	15	4	80	46	25	30	Sham

*Pain scores are on a 0–100 visual analog scale (VAS) with 0 being no pain and 100 worst pain imaginable*.

**Figure 1:**
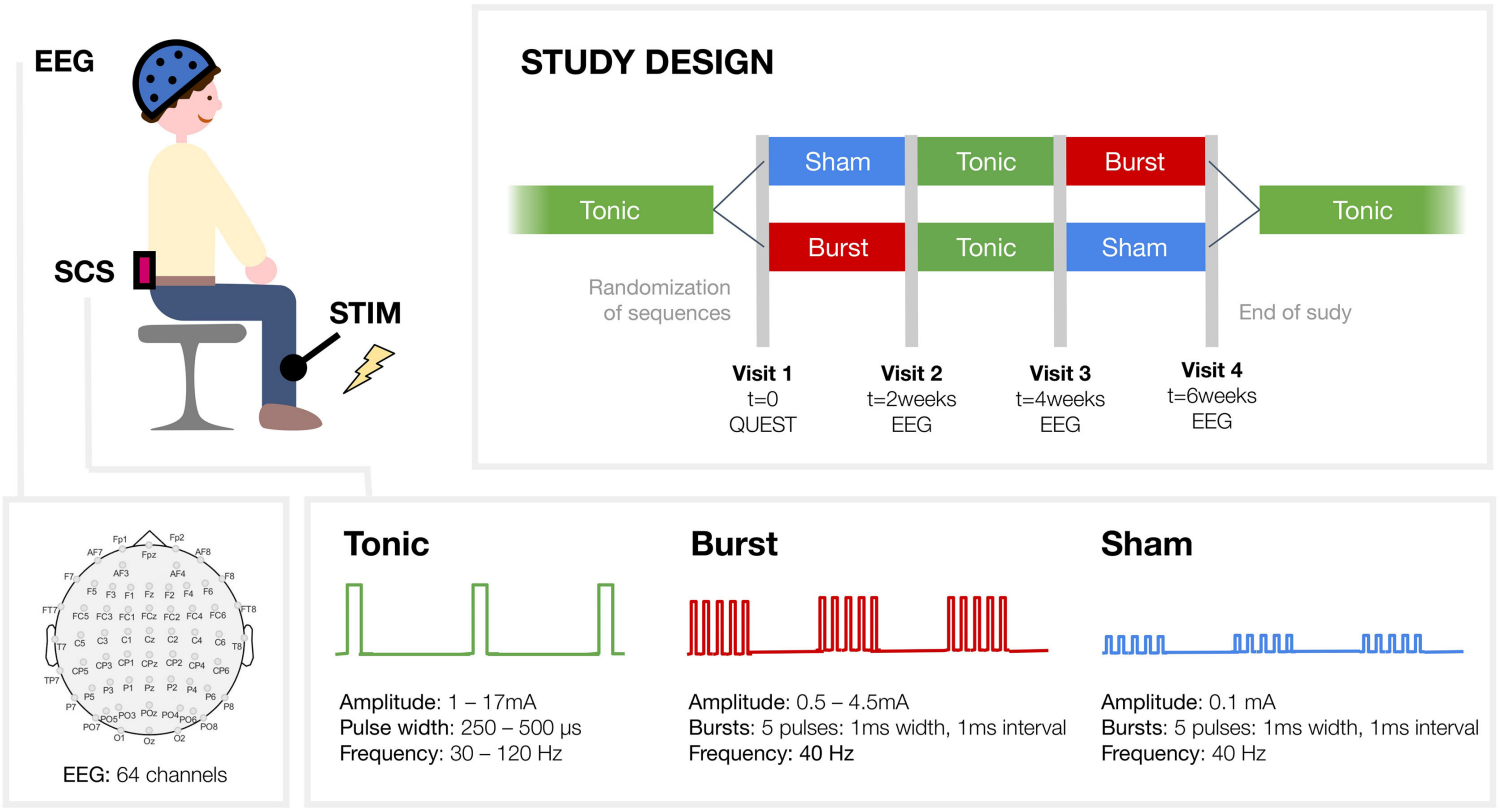
Study design. Patients were evaluating three different spinal cord stimulation (SCS) regimes, each for 2 week: conventional tonic SCS, burst SCS and sham SCS. Participants were randomized to one of the two study arms. Tibial nerve stimulation was applied to elicit somatosensory evoked potentials that were recorded with electro-encephalography (EEG). Two different conditions were studied: the attended condition, where participants had to silently count the administered tibial stimuli, and the unattended condition, where they were asked to mind wander.

All patients evaluated three different SCS settings, each for 2 weeks: conventional tonic SCS, burst SCS and sham SCS (Figure 1). Participants were randomized to either the “sham-tonic-burst” or “burst-tonic-sham” arm. Sham stimulation was a low amplitude burst stimulation intended to be non-therapeutically (19). However, the therapeutic range of burst stimulation was not known at the time this study was conducted and might be different for every patient. We can therefore not rule out that in some patients in the present study burst stimulation with 0.1 mA (very low amplitude) was indeed at a therapeutic level. Nevertheless, as this stimulation setting was expected to be non-therapeutically, in the present study we refer to the “low amplitude burst stimulation” setting as “sham stimulation.” Contrary to tonic SCS, both bust SCS and sham SCS are not sensed by the patient. So patients themselves knew when they received tonic SCS, but they did not know in which order they were evaluating burst or sham SCS.

### EEG Acquisition

Electroencephalography (EEG) measurements were conducted during three sessions: at visits 2, 3 and 4 (Figure 1). EEG data was acquired using a 64-channel Ag/AgCl EEG placed according to the extended international 10–20 system (Waveguard EEG cap, ANT Neuro, the Netherlands) see channel layout in Figure 1 and was recorded using ASA™ software (ANT Neuro, The Netherlands). The signals were amplified, low-pass filtered (digital FIR filter 1,350 Hz cut-off) and sampled at 5 kHz (TMSi-64 REFA, Twente Medical Systems International, the Netherlands). Impedance of the scalp electrodes was kept below 5 kOhm to reduce polarization effects.

### Tibial Nerve Stimulation

Patients sat comfortably in an armchair in an electrically and sound-shielded room. They received transcutaneous electrical stimulation to induce Somatosensory Evoked Potentials (SEP). Stimulation was applied to the tibial nerve at the ankle of their affected leg in order to study the evoked potentials in several cortical areas (Figure 1). Square wave pulses of 0.2 ms duration (constant current stimulator model DS7A, Digitimer Limited, UK) were delivered through surface electrodes, with the anode positioned distal, fixed over the tibial nerve. Per run 190 to 210 electrical pulses were given, with the exact number varying per run. Stimuli were delivered at an average frequency of 1.1 Hz, with the inter stimulus intervals randomly varying from 0.6 to 1.6 s. The stimulation amplitude was adjusted for the individual patient until a level that elicited a twitch of the big toe. In all cases the pulses could be clearly felt without being painful.

Non-painful electrical stimulation of the tibial nerve evokes the first positive EEG activity after about 40 ms (P40) in the primary somatosensory cortex. This activation is generally measured at the vertex (20). P40 activation is followed by the P60, which also reflects somatosensory processing in the foot area of the SI (15, 20). The subsequent negative activity N90 is believed to be generated in the somatosensory cortices and reflect sensory-discriminative processes (20–22). Then there can be a large broad increase in activity around 250 ms which is generally measured at the vertex as a response to attentional processing of stimulus events (23). This late activation around 250 ms has been shown to be particularly strong to painful somatosensory stimuli and is identified to reflect activity in the somatosensory and cingulate cortices (15, 24) and insular and opercular cortices (25, 26).

### Task

Each patient had three EEG recording sessions, one session with each of the three SCS regimes. During each session the spinal cord stimulator was still active with the same setting and intensity as it had been for the 2 weeks prior to the EEG recording. At each EEG session, a resting state EEG recording and two SEP recordings were made. Throughout the entire recording session the patients were asked to relax and keep their eyes open looking at a fixation cross. During one of the SEP recordings patients had to pay attention to the stimuli that were applied to their tibial nerve. They were asked to silently count them and afterwards report the number to the researcher. During the other SEP recording patients were asked to mind wander and not pay attention to the stimuli. The order in which stimuli had to be attended or not attended, was counterbalanced over the sessions and over the patients.

### EEG Analysis

#### Preprocessing

Power-line external noise on EEG signals was removed using a notch around 50 Hz and its harmonics. Data was bandpass filtered between 0.6 and 100 Hz (stopband attenuation 60 dB). Cardiac and blinking artifacts were also detected using the ECG and EOG signals and corrected using the Signal-Space Projection approach (27). All the EEG preprocessing and analysis was performed using Brainstorm (http://neuroimage.usc.edu/brainstorm/) (28) following the indications for group analysis suggested in Tadel et al. (27). Data was then visually inspected by a specialist to manually discard bad channels and remove noisy segments (on average, one channel (TP8) and <2% of data segments per subject).

#### Source Reconstruction

Participants head model was estimated using the Symmetric Boundary Element Method from the open-source software OpenMEEG: Scalp 1.0000 1082V | Skull 0.0125 642V | Brain 1.0000 642V) (29, 30). We used a default brain template [Colin27- a stereotaxic average of 27 T1-weighted MRI scans of the same individual, MNI brain with a 1 mm resolution (31)]. Full noise covariance matrix was computed based on the EEG recordings baseline period −200 to −4 ms for every subject. EEG source reconstruction was subsequently completed using the sLORETA approach [standardized LOw Resolution brain Electromagnetic TomogrAphy (32)] implemented in Brainstorm: loose 0.2, SNR 3, pca 1, diagnoise 0, regnoise 1, eegreg 0.1, depth 1, weightexp 0.5, weightlimit 10 and fixed source orientation, obtaining a surface of 15,000 vertices.

#### Somatosensory Evoked Potentials

Approximately 200 SEP epochs from −200 to 500 ms were averaged per participant, with time = 0 ms being the time of delivery of the electrical stimulation at the tibial nerve. Each epoch was DC offset corrected (i.e., for each signal, the mean of the baseline from −200 to −4 ms was computed, and then subtracted from each time sample) and the stimulation artifact [−4, 6 ms] was cut. Source data was Z-scored with respect to the baseline [−200, −4 ms] and individual cortical maps were smoothed using a circularly symmetric Gaussian surface kernel with a full width half maximum size of 10 mm (27). In order to compare our results with previous literature we obtain the SEP from a source defined at the vertex.

#### Statistics

Differences between SCS regimes (burst, tonic, sham) were evaluated on the different SEP amplitudes at latencies of interest at the vertex (i.e., 40, 60, 90, and 250 ms) using two sided non parametric Wilcoxon signed rank tests and a two-way analysis of variance (ANOVA) test, including SCS regime (burst, tonic, sham) and participants attention condition (attended, unattended). In addition, whole brain source differences between conditions were estimated correcting for multiple comparisons using a non-parametric cluster based permutation test (paired *t*-test, 1,000 permutations, *p* < 0.05, cluster alpha 0.05) (33).

## Results

### Subjects

Clinically, the 12 participants responded differently to the three spinal cord stimulation regimes they evaluated. The amount of pain relief by the three different SCS regimes varied largely for the individual patients. The average VAS scores for pain were 46 (range: 17–79) for burst SCS, 61 (range: 17–90) for tonic SCS, and 53 (range: 21–72) for sham SCS (Table 1). Six patients preferred burst SCS, four patients preferred tonic SCS, two patients preferred sham SCS. Preference was mainly based on the largest pain relief, but was also influenced by the perception of the paresthesia: five patients liked the absence of paresthesia, while one patient mentioned he really missed the paresthesia sensations, and two patients missed reassurance that the stimulation was active and that they could feel changes in stimulation intensity when they used their remote control. Both sham and burst SCS were not sensed by any the patients, while all of them could feel at least some paresthesia with tonic SCS. During the study, patients did not know in which phase they had sham SCS or burst SCS.

### Somatosensory Evoked Potential

All participants could clearly feel and count the applied stimuli at their tibial nerve in all three SCS conditions. Average stimulation amplitude was 27 mA (range: 10–75 mA). The individual's stimulation amplitude was stable over the three EEG recording sessions (<10% variation for each subject) and so was the perceived intensity of the applied stimuli, which was non-painful but clearly sensed by all participants. All participants were therefore able to focus on the applied stimuli and could count them correctly, with an average accuracy of 98% for all three SCS conditions. Since none of the participants perceived the stimuli as painful in either of the three conditions, all reported that they were capable of shifting their attention away from the stimuli during the trials in which they were asked to mind wander.

Neither attention nor SCS setting had a statistically significant effect on the amplitudes of the early SEP latencies (P40, P60, N90). Which corresponds with the similar intensity scores that were reported for the applied stimuli by the participants during all three SCS settings.

#### Effect of Attending the Applied Stimuli

**Figure 2:**
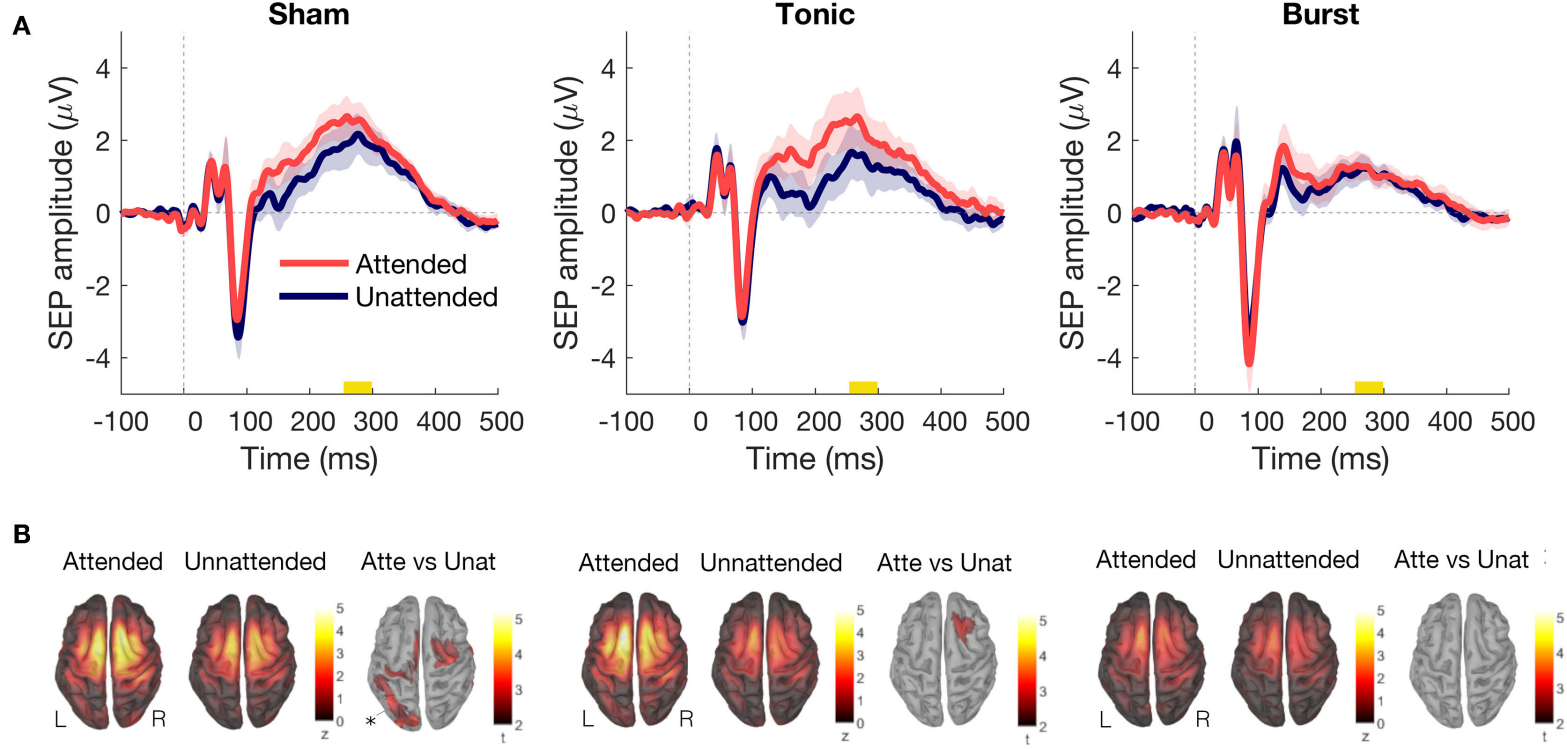
**(A)** Difference in somatosensory evoked potential between attended (red) and unattended (blue) conditions on the vertex for each type of spinal cord stimulation (SCS): sham SCS (left), tonic SCS (middle), and burst SCS (right). Shadows indicate standard error of the mean (200 epochs per condition, *n* = 12 subjects). **(B)** P300 source activation (from 250 to 300 ms), for each type of SCS and condition. Statistical results with non-parametric cluster based permutation tests. All results showed Attended > Unattended.

Sham SCS was intended to have no therapeutic effect. Attending the electrical stimuli applied at the tibial nerve during sham SCS caused no statistically significant differences in P300 amplitude as when the stimuli were not attended (Figure 2A). Comparing the evoked activity in time window 250–300 ms between the attended and unattended condition revealed increased activity in the right somatosensory, motor and cingulate cortices, and in occipital and temporal areas during attention (Figure 2B).

When the participants were asked to mind wander during tonic SCS and did not attend the applied stimuli, the P300 amplitude decreased substantially in comparison to the attended condition (Figure 2A). The right prefrontal cortex showed significantly more activity in the attended condition as compared with the unattended condition (Figure 2B).

During burst SCS, paying attention to the applied stimuli or not attending them did not change the P300 amplitude of the SEP (Figure 2).

#### Effect of Spinal Cord Stimulation Setting

**Figure 3:**
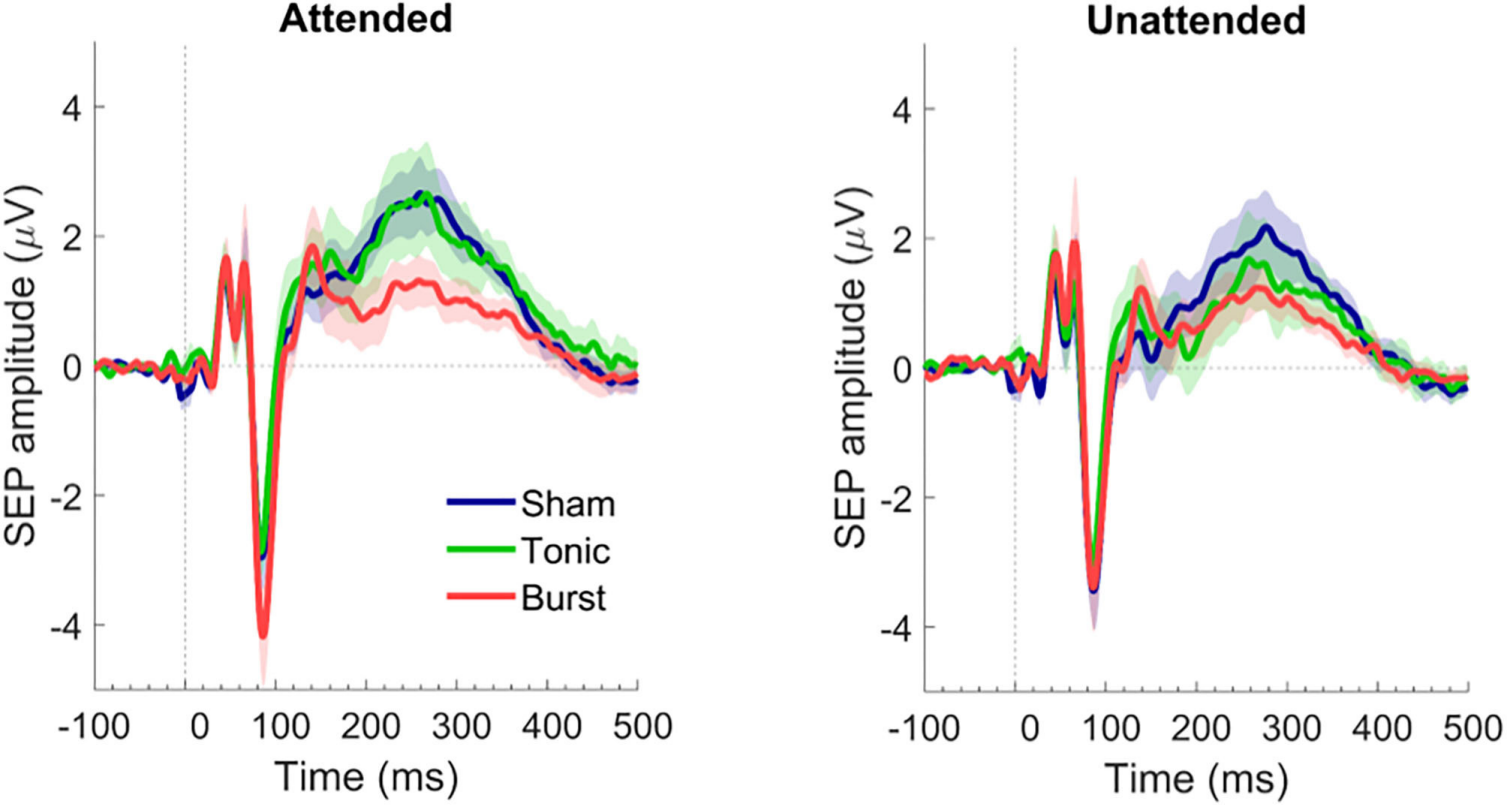
Somatosensory evoked potential in attended **(left)** and unattended **(right)** conditions on the vertex for each type of spinal cord stimulation: sham SCS (blue), tonic SCS (green), and burst SCS (red). Shadows indicate standard error of the mean (200 epochs per condition, *n* = 12 subjects).

When the applied stimuli were attended by the participants, only the burst SCS regime reduced the P300 amplitude as compared with sham SCS (Figure 3). When the stimuli applied to the tibial nerve were not attended, because the participants were mind wandering, both the tonic and the burst SCS regime reduced the P300 amplitude as compared to sham SCS, with the lowest P300 amplitude during burst SCS (Figure 3). The largest difference, however, between the attended and unattended condition was obtained under tonic SCS regime (Figure 2A).

**Figure 4:**
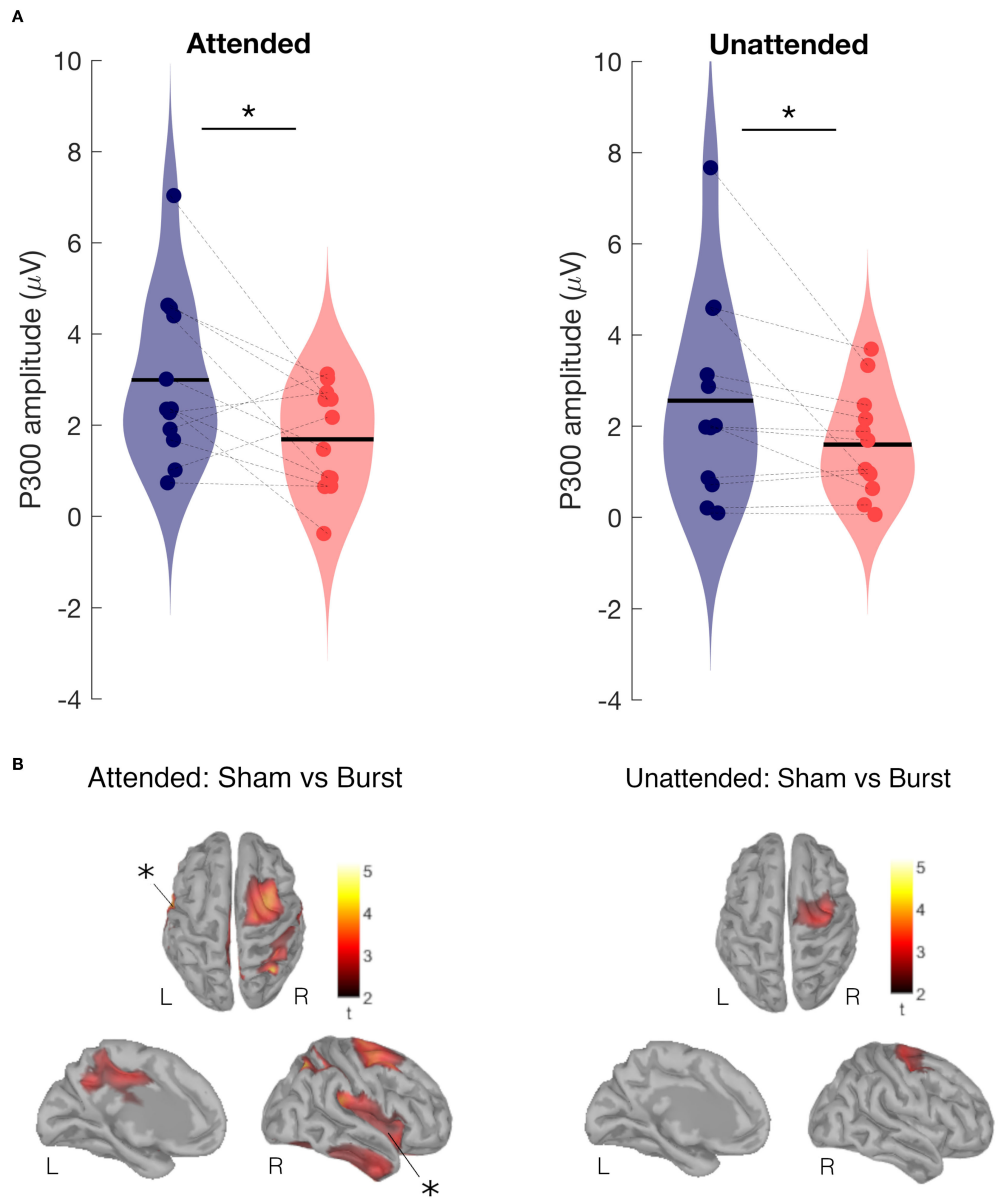
Attended and unattended condition. **(A)** P300 amplitude of SEP at the vertex during burst SCS (red) and during sham SCS (blue). Two-sided Wilcoxon signed rank test, *p < 0.03. **(B)** Effect of burst vs. sham SCS on brain sources averaged for the P300 period (250 to 300 ms), cluster based permutation test on the absolute values (paired *t*-test, 1,000 permutations, cluster alpha 0.05). Top view (top), left hemisphere internal view (left), and right hemisphere (right). Results show Sham>Burst in right supplementary motor area, somatosensory cortex, mid cingulate cortex, and left and right insular cortices. Statistically significant results are indicated with *.

In both the attended and unattended condition, the amplitude of the P300 was significantly smaller (*p* < 0.03) during burst SCS in comparison to sham SCS (Figure 4). In the attended condition this was the case for all latencies from 200 to 300 ms after the electrical pulse was applied to the tibial nerve. No significant differences were found between tonic and sham SCS.

Comparing at source level, the evoked activity in the time window 250–300 ms between burst SCS and sham SCS revealed decreased activity during burst SCS in the right supplementary motor area (SMA), somatosensory, mid cingulate, and left and right insular cortices (Figure 4B).

## Discussion

Attention can modulate the amplitude of the P300 in chronic pain patients treated with spinal cord stimulation. When patients either silently counted the applied electrical stimuli or were mind wandering, this modulated the SEP amplitude of the P300, but not the evoked potentials at earlier latencies and neither the perceived intensity of the applied stimuli.

Previous findings by De Ridder et al. (12) and De Ridder and Vanneste (13) have related the analgesic effect of burst spinal cord stimulation to reduced attention to pain. If burst SCS indeed reduces the capacity for attention to be directed to pain, then this should be reflected in reduced amplitudes of the attention-related component of the SEP, the P300 (23). In this explorative study, we have measured SEPs in 12 chronic pain patients who had 2-week evaluation periods with conventional tonic SCS, burst SCS and sham SCS, and who did not know at which moment they had sham SCS or burst SCS. When patients were asked to actively attend the electrical stimuli applied at their tibial nerve, there was a significant reduction in P300 amplitude when patients were under burst SCS regime as compared with sham and tonic SCS regimes (Figure 3). Even though both burst and sham SCS settings were not sensed by the patients, and patients did not know which of those two SCS regimes was active at the moment of the measurement.

During sham SCS, attending or not attending the peripherally applied stimuli only caused a small difference in P300 amplitudes, indicating that even while the patients were mind wandering, somatosensory stimuli were still processed to almost the same amount as if they were attended. Attending the applied stimuli while receiving tonic SCS showed a P300 amplitude similar to the sham conditions. However, when the stimuli were not attended, the amplitude decreased substantially. During burst SCS, not attending the stimuli caused an even lower P300 than not attending them during tonic SCS. Attending the stimuli during burst SCS however, did not cause an increase in the P300 amplitude, indicating that burst SCS might affect the attention that could be directed to the applied somatosensory stimuli.

### Subjects

The 12 participants in this study are not healthy subjects, but chronic pain patients who have been suffering neuropathic pain for years and are treated with analgesic medication and SCS therapy. SEP amplitudes and latencies in chronic pain patients vary from healthy subjects, as amplitudes have been reported larger and certain latencies to be delayed (34). Therefore, in this study, aimed to specifically assess the effects of both conventional and burst SCS therapy on somatosensory processing, the participating patients were their own controls and we had one condition with sham SCS, which was intended to be a non-therapeutic SCS intensity.

Besides SCS therapy, most of the patients were also using analgesic medication. Although high dose opioids can induce an increase in low frequency (delta band) brain activity and a decrease in amplitude of potentials evoked by painful stimuli, opioids have been reported to not influence the amplitudes of the non-painful SEPs (35). Paracetamol and pregabalin have been reported to not alter painful SEPs (36, 37). NSAIDs have been reported to alter amplitudes and latencies of painful SEPs, but there are no reports on their influence on non-painful SEPs (35). Even if analgesic medication has influenced the SEP in some participants, it has done that in equal amounts for all three SCS conditions, as none of the patients changed their dose or type of medication over the course of this study.

### Spinal Cord Stimulation

In our study, the participants had their spinal cord stimulator for at least half a year, so all of them were familiar with tonic SCS and the paresthesia it caused. This means we have a different population than De Ridder et al. reported on (12, 13). Their five patients were completely new to SCS and still trialing SCS with an external pulse generator. The sensation of (tonic) SCS was new to the participants in the study by De Ridder et al., while for our patients it was the absence of sensations during sham and burst SCS which was a new experience. We have therefore conducted our SEP measurements after 2-weeks evaluation periods with burst SCS and sham SCS, with a 2-weeks period with tonic SCS in between the burst and the sham SCS.

For each participant the amplitude and the perceived intensity of the applied tibial nerve stimuli did not vary during the three study conditions. However, the effects of the three SCS regimes (burst, tonic, and sham) on their own ongoing pain varied largely over the participants, as did their preference for specific SCS settings. Preference was also influenced by other aspects than pain reduction, like the presence or absence of paresthesia (19). The number of participants in the present study is too small to separately analyze effects by either preference or pain relief. Therefore, effects that we report on brain activity are independent of the clinical effects of the SCS regimes.

### Somatosensory Evoked Potential

To make sure that the participants actively attended the applied electrical stimuli at their tibial nerve, we asked them to silently count the stimuli and report the number at the end of the measurement. To avoid them just remembering the number of applied stimuli from previous measurements, we randomly varied the number of stimuli of every SEP recording. Since the patients reported the correct numbers in all attention conditions, it is very likely they were indeed counting and paying attention. In the mind wandering condition, we can never be completely certain that patients were indeed not counting or otherwise attending the stimuli when we asked them not to do so. However, the differences in P300 amplitude between the attended and unattended stimuli in the sham and tonic SCS conditions suggest that patients were again compliant and were not attending the stimuli during these recordings.

Tonic SCS is accompanied by paresthesia, generally by the patients described as constant tingling sensations. When we applied electrical stimuli to the tibial nerve, these electrical stimuli and the paresthesia are concurrent sensations that need to be processed by the brain, in addition to the patient's ongoing pain. When the patients were asked to not attend the applied stimuli under tonic SCS, it led to a decreased P300 amplitude and reduced activity in somatosensory and motor cortices (Figure 2), which did not happen during sham or burst SCS regimes. The concurrent processing of paresthesia seems to allow the participants to pay less attention to somatosensory stimuli when they are asked to.

Other studies have found that conventional tonic SCS inhibits the early SEP latencies that are generated in the primary somatosensory cortex (18, 38). One case report even showed complete inhibition of the early SEP during conventional tonic SCS as well as during high frequency SCS and high density tonic SCS (17). We have not found complete inhibition nor statistically significant decreases in early peaks with either burst or tonic SCS as compared to sham SCS. However, our sham SCS was probably not at a subtherapeutic intensity for every participant, so there is a chance that all three SCS regimes reduced the early SEP amplitudes to the same amount. Still, our Figures 2, 3 show early latency peaks P40, P60, and N90 with amplitudes similar to the “no stimulation” conditions reported previously (17, 38). No statistical differences in early amplitudes between the SCS regimes or attention conditions were found in the present study.

One other study also reported reduced late SEP (P300) amplitudes in response to non-painful tibial nerve stimulation during tonic SCS as compared with no SCS (15). In that study, participants were not specifically asked to attend or not-attend the applied stimuli and the SEP was obtained directly after tonic SCS was switched ON or OFF. Therefore, it is difficult to disentangle the effects of the different conditions and it is possible that some participants were attending the stimuli while others were not. In addition, the effects of the previous SCS setting might not have ceased completely when they already did their next measurements, which could explain the smaller amplitude difference in their results. The time period during which SCS effects maintain after a setting has been changed, can vary largely among patients and can last up to hours for some individuals (39). Therefore, in the present study, the SEP measurements were conducted at the end of the 2-weeks evaluation period of an SCS regime.

Polácek et al. (15) applied source dipole fitting on their SEP data and calculated the main origin of their P300 at the midcingulate cortex. The P300 peak is believed to consist of an earlier component P3a (generated in frontal areas) and a later component P3b (generated in temporal-parietal areas). The P300 (P3a) and activation of the mid cingulate cortex is larger in amplitude when a stimulus is novel and attentional focus is oriented to sensory stimuli (23, 40). In the P300 latency range, we find differences between burst SCS and sham SCS in activation, not only in the mid cingulate cortex, but also in insular cortex, somatosensory cortex and SMA (Figure 4B). Similar areas show differences in activation when comparing the attended condition with the unattended condition during sham SCS (Figure 2B). Attention to pain tends not only to increase the perceived intensity of pain, but also the magnitude of the insular activity. The insula plays a role in the detection of salient stimuli and modulation of the reaction to these stimuli (40, 41). Decreased activity in mid cingulate and insular cortices during burst SCS as compared with sham SCS suggests that the salience network is less engaged when a patient receives burst stimulation. In addition, we found decreased activity with burst SCS in the supplementary motor area and somatosensory cortices, which are part of the dorsal attentional network that is involved in the top-down selection of which stimuli are attended and how to respond to them.

### Limitations

Our EEG study is an explorative study, with a small number of participants to search for potential differences in effects and working mechanisms between tonic and burst spinal cord stimulation. In addition we compared burst and tonic SCS with sham SCS. Only 12 chronic pain patients with an implanted SCS device participated in our study that was underpowered. Interpreting the statistical results has to be done carefully, but since the effects of tonic and burst SCS happen in those 12 patients regardless of the effect of SCS on their pain, our results are interesting to further test in an properly powered study.

A major limitation of our present study, however, is that sham SCS might not have been real sham stimulation for every participant and some patients might have actually received sufficient energy to perceive therapeutic effects. Two patients reported their lowest pain scores under sham SCS regime. The therapeutic range of burst SCS is still unknown and this range might vary to a great extent per patient, similar to how the therapeutic range of conventional tonic SCS varies per patient and is among others dependent on the individual's anatomy of the spinal cord and the position of the electrode lead in the epidural space (42, 43). However, defining the therapeutic range of burst is largely complicated by the fact that burst SCS is not sensed, which hinders (direct) feedback from the patient. Studies conducted after we collected our data indicate that the therapeutic range of burst SCS might go as low as 0.1 mA for individual patients (44).

## Conclusion

Burst stimulation is one of the relatively new developments in spinal cord stimulation regimes. Several aspects of the working mechanisms of burst stimulation and other new paresthesia-free regimes are still unknown and require further research, as they seem to affect different or additional cortical areas than tonic SCS. The present study showed that burst SCS reduced the P300 amplitude of the somatosensory evoked potential. A similar reduction was also obtained during tonic SCS when patients were instructed to not attend peripherally applied pulses. Which suggests that burst SCS reduced the capacity for attention directed to somatosensory stimuli.

Our findings support the hypothesis posed by De Ridder and Vanneste (13) that burst SCS modulates activity in pain processing brain areas in a similar manner as when somatosensory stimuli are not attended. This effect of burst SCS was present in general, even when the participants were instructed to pay attention to the applied somatosensory stimuli. Overall, burst SCS acted without reducing the perceived intensity of the peripherally applied stimuli and regardless of the analgesic effect of burst SCS on the patient's own pain. In conclusion, burst spinal cord stimulation for the treatment of chronic pain seems to reduce the attention that is or can be directed to somatosensory stimuli, probably to a larger extent than conventional tonic spinal cord stimulation treatment. This is a first step in understanding why in selected chronic pain patients burst SCS is more effective than tonic SCS and how neuroimaging could assist in personalizing SCS treatment.

## Data Availability Statement

The data and code supporting the conclusions of this article will be made available by the authors, without undue reservation.

## Ethics Statement

The studies involving human participants were reviewed and approved by Twente Ethics Committee, Enschede, the Netherlands. The patients/participants provided their written informed consent to participate in this study.

## Author Contributions

The conception was originated from CdV and ML. Data collection was done by MT-C and CdV. Data analysis and interpretation was performed by GN and CdV. The first draft was written by GN and CdV and further input was provided by ML and MT-C. All authors contributed to the article and approved the submitted version.

## Conflict of Interest

The authors declare that the research was conducted in the absence of any commercial or financial relationships that could be construed as a potential conflict of interest.

## Publisher's Note

All claims expressed in this article are solely those of the authors and do not necessarily represent those of their affiliated organizations, or those of the publisher, the editors and the reviewers. Any product that may be evaluated in this article, or claim that may be made by its manufacturer, is not guaranteed or endorsed by the publisher.
